# Functional traits provide new insight into recovery and succession at deep‐sea hydrothermal vents

**DOI:** 10.1002/ecy.3418

**Published:** 2021-07-02

**Authors:** Lauren N. Dykman, Stace E. Beaulieu, Susan W. Mills, Andrew R. Solow, Lauren S. Mullineaux

**Affiliations:** ^1^ Woods Hole Oceanographic Institution Woods Hole Massachusetts 02543 USA

**Keywords:** benthic invertebrates, disturbance, functional traits, hydrothermal vents, recovery, succession

## Abstract

Investigation of communities in extreme environments with unique conditions has the potential to broaden or challenge existing theory as to how biological communities assemble and change through succession. Deep‐sea hydrothermal vent ecosystems have strong, parallel gradients of nutrients and environmental stress, and present unusual conditions in early succession, in that both nutrient availability and stressors are high. We analyzed the succession of the invertebrate community at 9°50′ N on the East Pacific Rise for 11 yr following an eruption in 2006 in order to test successional theories developed in other ecosystems. We focused on functional traits including body size, external protection, provision of habitat (foundation species), and trophic mode to understand how the unique nutritional and stress conditions influence community composition. In contrast to established theory, large, fast‐growing, structure‐forming organisms colonized rapidly at vents, while small, asexually reproducing organisms were not abundant until later in succession. Species in early succession had high external protection, as expected in the harsh thermal and chemical conditions after the eruption. Changes in traits related to feeding ecology and dispersal potential over succession agreed with expectations from other ecosystems. We also tracked functional diversity metrics over time to see how they compared to species diversity. While species diversity peaked at 8 yr post‐eruption, functional diversity was continuing to increase at 11 yr. Our results indicate that deep‐sea hydrothermal vents have distinct successional dynamics due to the high stress and high nutrient conditions in early succession. These findings highlight the importance of extending theory to new systems and considering function to allow comparison between ecosystems with different species and environmental conditions.

## Introduction

Disturbance is a ubiquitous and important driver of biological patterns in most ecosystems. An understanding of the processes influencing the pattern of succession following disturbance has been the focus of a century of research (e.g., Clements 1916, Gleason 1926, Egler 1954, Connell and Slatyer 1977, McCook 1994) and is of growing practical importance with the recent unprecedented rate of anthropogenic habitat destruction and alteration (Sasaki et al. 2015). While a primary goal has been to identify general patterns of succession (Clements 1916, Odum 1969), the complex interplay of biotic and abiotic factors makes such generality difficult. These factors include site condition (Berlow 1997), the type and supply of initial colonists (Egler 1954, Sutherland and Karlson 1977), niche preemption and modification by colonists (Jones 1997, Fukami 2015), and post‐colonization species interactions (Connell and Slatyer 1977, Farrell 1991). In many successional systems, physical conditions and resources change over time and drive shifts in community structure by acting on the physiological and nutritional requirements of individual species (Tilman 1985).

The environment at deep‐sea hydrothermal vents differs chemically, physically, and nutritionally from most well‐studied successional systems. The hydrothermal fluids reach temperatures of 400°C and, when undiluted by ambient seawater, exhibit low pH, low oxygen, and concentrations of hydrogen sulfide and other chemicals that are toxic to animal life (Childress and Fisher 1992). These reduced chemicals, however, also provide the energy for microbial chemosynthetic production of the organic matter that fuels the food web. As venting fluid exits the seafloor and mixes with ambient seawater, a gradient is established over which environmental stressors and nutrients vary in parallel (Micheli et al. 2002). Covariance of stress and nutrients is likely an important feature of succession at vents because the source of large‐scale disturbance at vents, seafloor eruptions, provides an environment for initial colonists that is physiologically stressful yet also enriched in nutrients in the form of reduced chemicals. This is opposite the trend observed in classic examples of primary succession (i.e., the rocky intertidal, volcanic islands), where the highest abiotic stress (desiccation, limited shelter, high thermal stress) generally co‐occurs with the lowest resource availability directly after disturbances (Connell 1961, Tsuyuzaki and del Moral 1995). Thus, for aspects of species succession that are influenced by physiochemical stress and/or nutrition (e.g., body size, physical protection, trophic position), patterns at vents may differ from those in other systems in ways that challenge or broaden existing theory.

Hydrothermal vents on the East Pacific Rise (EPR) are subject to frequent large‐scale volcanic disturbance, providing repeated opportunities to study primary succession in the deep sea. The EPR segment between 8° and 10° N is categorized as a fast‐spreading center (80–150 mm/yr) (Bird 2003) and experiences eruptions on a decadal timescale (Rubin et al. 2012). Early stages of succession have been tracked at the 9°50′ N vent field on the EPR after two eruptions, one in 1991 (Haymon et al. 1993) and one in 2006 (Tolstoy et al. 2006), which paved over existing invertebrate communities with basaltic pillow lava. Venting flux was vigorous and concentrated following both eruptions, but declined and became more diffuse over time, with a corresponding increase in pH and decrease in reduced sulfur concentration (Le Bris et al. 2006). Visual surveys in the first 3–4 yr following both eruptions showed a repeated pattern of initial microbial colonization, followed by sequential settlement of two different siboglinid tubeworms (*Tevnia jerichonana* and *Riftia pachyptila*) and then mussels (*Bathymodiolus thermophilus*) (Shank et al. 1998, Fornari et al. 2012). Although species assembly patterns have been documented at 9°50′ N for early succession (Shank et al. 1998), a comparison with general theory requires longer tracking and consideration of the function, as well as the identity, of the full size‐spectrum of invertebrate colonists.

To allow comparison of successional patterns involving systems with distinct conditions and species assemblages, ecologists have used the notion of functional traits (Meiners et al. 2015). The idea is that, by considering the functional role of species rather than just their specific taxonomy, we can gain insight into how organisms affect and respond to features of their environment (Cadotte et al. 2011, Mouillot et al. 2013). Functional trait analysis has proven useful in plant (Reich et al. 1997), insect (Ding et al. 2017), and microbial ecology (Zak et al. 1994), and has recently expanded into oceanic systems (Micheli and Halpern 2005, Aguilera and Navarrete 2012, Darling et al. 2012, Stuart‐Smith et al. 2013, Teixidó et al. 2018). Here, we use an unprecedented 11‐yr colonization time series initiated after the 2006 eruption at the 9°50′ N hydrothermal vent field on the East Pacific Rise, combined with a growing trait database for hydrothermal vent invertebrates (sFDvent; Chapman et al. 2019), to test trait‐based successional hypotheses in a new setting with the unusual conditions associated with deep‐sea eruptions (i.e., high stress and high nutrients in early succession).

We examine a suite of traits and functional diversity metrics that have been observed to change over succession in other marine benthic systems (Bolam et al. 2016, Greenfield et al. 2016, Veríssimo et al. 2017). We explore whether some of these trait patterns diverge from theory, specifically those related to growth, acquisition of nutrients, and provision of habitat. In non‐vent systems, where nutrients typically are depleted after a disturbance, small‐bodied species colonize first, and biogenic structure (habitat complexity) increases over time (Tilman 1988, Hirata 1992, Teixidó et al. 2004). At vents, where a pulse in nutrients occurs during the disturbance, we expect that large‐bodied species will be able to grow and create habitat complexity early in succession, thus facilitating the rapid assembly of diverse trophic levels and feeding modes. Later in succession, when venting (and thus nutrients) becomes more diffuse and less reliable, animals with higher mobility may be favored. Other trait patterns are expected to follow established theory. Species with greater external protection are likely to be more abundant in early succession when physiological stressors are highest. Animals with high dispersal potential are likely to colonize earliest, as observed in other patchy, island‐like habitats (MacArthur and Wilson 1967, Tsuyuzaki and del Moral 1995, Thornton 2007), and asexually reproducing organisms, if able to arrive early, will rapidly establish. In addition to traits, we track functional diversity indices over the course of succession to see whether they provide additional information compared to species diversity indices. We discuss the application of these results to vent ecology and conservation, as well as the new insight gained from expanding successional theory to ecosystems with atypical conditions.

## Materials and Methods

### Biological samples

**Fig. 1:**
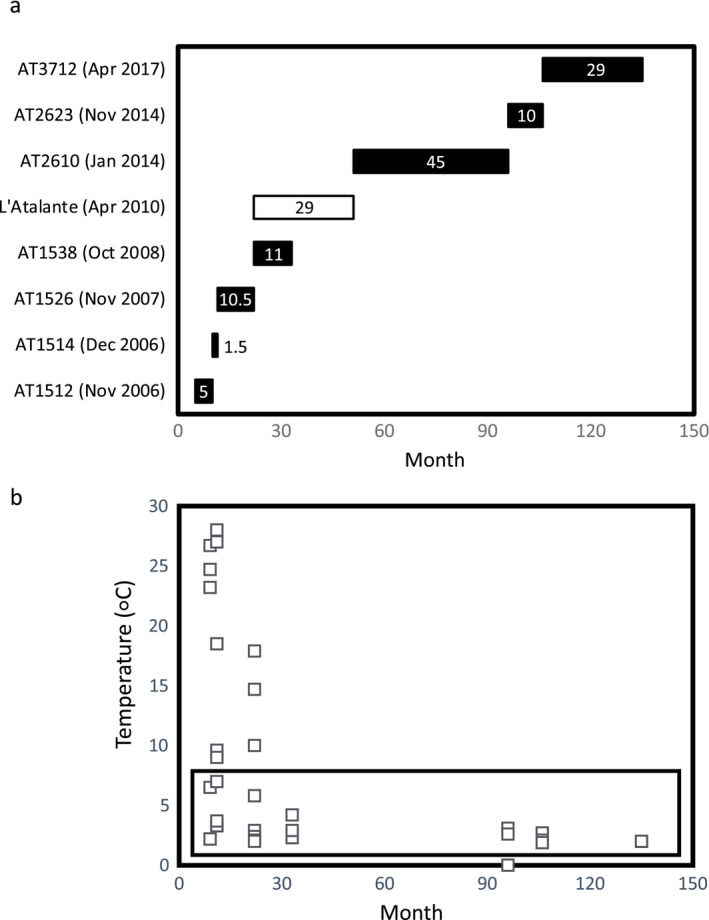
(a) Deployment durations of colonization sandwiches in months after the 2006 eruption, labeled by the *Atlantis* or *L’Atalante* recovery cruise. Numbers in the bars indicate deployment duration in months. No data were available for the *L’Atalante* cruise (white bar) as sandwiches were coated with ferrous precipitate. (b) The temperature measured at the base of each sandwich at the time of recovery. The 1.9°–6.5°C subset of samples included in analysis are within the black box.

The post‐eruption colonization samples examined for this study were collected from 9°50′ N on the EPR at seven time points between November 2006 and April 2017. Sampling focused on P‐vent (9.8380° N, 104.2912° W, 2,509 m depth), where the January 2006 eruption had destroyed the local invertebrate community. Colonists were collected from introduced samplers, called “sandwiches” (Appendix S1: Fig. S1), as described in Mullineaux et al. (2010). Sandwiches were deployed by the deep submergence vehicles *Alvin* and *Jason* across the range of temperatures representative of the environmental and biological zones of the vent field (Micheli et al. 2002). Sandwiches were left on the seafloor for varying intervals between six weeks and four years, based on cruise availability (Fig. 1a), and new sandwiches were deployed in the same location as recovered ones, resulting in a continuous time series of colonist abundance.

To characterize the fluid environment at each sandwich, temperature was measured at deployment and recovery by holding a temperature probe at the base for 1–2 minutes until a definite maximum was reached. We used recovery temperature to characterize the thermal environment for subsequent analyses because it reflected the most recent conditions experienced by the community on the sandwiches at the time of collection. From the full set of post‐eruption colonization samples, we selected 30 with recovery temperatures of 1.9°–6.5°C, as this was the thermal range adequately sampled throughout the full observation period (Fig. 1b). Temperatures in the vent field had generally waned over time (Mullineaux et al. 2010), so samples with recovery temperatures greater than 6.5°C were not available after 22 months. This subsampling was necessary to minimize the influence of environmental change, as many vent species have narrow thermal and chemical preferences (Childress and Fisher 1992), while retaining three to six sandwiches for each time point.

Six pre‐eruption samples (recovery temperatures 1.9°–6.5°C) that had been collected in 1998 after a 37‐month deployment at the nearby site East Wall (9.8421° N, 104.2919° W, 2,506 m depth) were also included in the analysis. These samples were basalt colonization blocks rather than sandwiches; however, previous analyses indicate that blocks and sandwiches collect similar colonists (Mullineaux et al. 2010). East Wall was the best available example of a mature community close to P‐vent, though the fauna sampled at East Wall may not represent a traditional “climax community” because the site had experienced a prior eruption in 1991 (Shank et al. 1998), 85 months before the sampling.

Sandwiches and blocks were brought to the surface in separate, sealed collection compartments and preserved in 80% ethanol, along with any specimens that fell off in the collection compartments. For each sandwich or block, specimens were visually inspected under a dissecting microscope and identified morphologically to species or the lowest possible taxonomic level. Species‐level identification of many juveniles was possible by comparing morphology to adults that retain larval characteristics (Adams et al. 2010). Individuals that were too young to identify to species were grouped by genus or family. For this reason, some individuals of a species might have been categorized differently depending on size (e.g., *Lepetodrilus elevatus* as *Lepetodrilus* spp., *Tevnia jerichonana* as siboglinids). This analysis includes metazoan invertebrates found on sandwiches or blocks, plus individuals found in the collection compartment that were retained on a 1 mm sieve. Counts of individuals per species or higher taxon for each of the 36 sandwiches or blocks used in this analysis (both pre‐ and post‐eruption) are provided as Data S1: CountsPerSpeciesPerSandwich_EPR.csv, and as part of the full sample set (Mullineaux 2020, 2020a). Colonist abundances were pooled across all sandwiches or blocks from each recovery date for subsequent analyses.

Of the 68 metazoan invertebrate species or morphotypes encountered, 58 had sufficient trait data to be included in this analysis. *Lepetodrilus* spp. and siboglinid spp. (formerly known as vestimentiferans) were too young to identify to species. These groups were retained because they were abundant in samples and have been identified as important participants in successional interactions (Shank et al. 1998, Mullineaux et al. 2009). Based on genetic sequencing of a subset of individuals, most individuals in the group *Lepetodrilus* spp. are likely *Lepetodrilus tevnianus*, and most individuals in the group siboglinid spp. are likely *Tevnia jerichonana* (Appendix S2).

### Biological traits

Eight traits were chosen to test our successional hypotheses and explore a range of ecological effects and responses (Table 1). Here, we define “trait” as a feature or behavior of a species that affects or responds to its environment, and “modality” as a scoring level reflecting how the organism expresses a given trait. Four traits were taken from the sFDvent Database: maximum adult body size, habitat complexity, trophic mode (i.e., trophic level), and relative adult mobility (Appendix S3). Four additional traits were added due to their ecological relevance and common use in functional analyses for aquatic invertebrates. These are external protection, feeding method (i.e., how a species feeds), reproductive type, and larval development (Bolam et al. 2016, Greenfield et al. 2016, Veríssimo et al. 2017). For each trait, a modality was assigned for every species based on literature or personal observation. For the traits from sFDvent, modalities were assigned from the recommended data set, except in cases where we suggested updates (Appendix S3: Table S1). When species identity was uncertain and individuals were identified to a higher taxonomic level (e.g., amphipods), modalities were assigned from a likely species that is found at our site and included in sFDvent. For the four traits not included in sFDvent, we either provided a citation or cited “expert opinion,” meaning the modality choice was based on direct observation by one of the co‐authors. Although the majority of specimens in our study were juveniles, modality assignments were based on the characteristics of adult organisms due to the availability of data. Modality assignments were fixed for a given species rather than specific to life stages or individuals (Data S1: ModalitiesPerTraitPerSpecies_EPR.csv; Dykman et al. 2021). For analysis, the abundance of a modality within a trait was taken as the total number of individuals expressing the modality.

**Table 1:** The eight selected traits and their modalities, including expected change over succession.

Trait	Trait type	Modality	Expected change over succession
Maximum adult body size	ordered	small (~1 mm) medium (~10 mm) large (~100 mm) very large (~1,000 mm)	Unlike in other systems, where early‐successional species are generally small (Odum 1969), large animals will colonize early at vents.
Habitat complexity	categorical	does not add complexity mat forming (<10 cm) bed forming (>10 cm) open bush forming dense bush forming	Unlike in other systems, where structure forms slowly (Tilman 1988, Hirata 1992, Teixidó et al. 2004), structure‐forming species will colonize early at vents (Shank et al. 1998).
Trophic mode	ordered	symbiont bacterivore detritivore carnivore S (scavenger) carnivore O (other)	As in other systems, low trophic levels will be abundant in early succession, and a greater number of higher trophic levels will assemble over succession (Margalef 1963, Odum 1969, Boit and Gaedke 2014). We expect the assembly of trophic diversity will progress rapidly at vents due to the high nutrient input.
Feeding method	categorical	non‐feeding deposit feeder suspension feeder predator parasite or commensal	As in other systems, symbiont hosts and microbial mat grazers will colonize early when microbial production is highest, whereas suspension feeders, scavengers, parasites, and predators will colonize later. We expect this transition to occur rapidly at vents.
Relative adult mobility	ordered	sessile movement restricted crawler freely mobile	Greater mobility will be favored in late succession when venting flux is less vigorous and more diffuse.
External protection	ordered	soft bodied moderately protected well protected	As in other systems (Connell 1961), animals will have more external protection in early succession, when environmental stressors are highest. Decreased stress and increased biogenic structure in late succession will facilitate soft bodied organisms.
Larval development	categorical	lecithotrophic planktotrophic brooding direct	As in other patchy, island‐like systems, we expect species with limited dispersal potential (brooding and direct development), will not establish until later in succession (Tyler and Young 1999).
Reproductive type	categorical	gonochoristic hermaphroditic asexual	As in other systems, we expect asexual organisms will be most abundant in early succession, as this is a strategy for rapid population growth (MacArthur and Wilson 1967).

Notes

Modality assignments and references per trait for each species are provided in Data S1: [ModalitiesPerTraitPerSpecies_EPR.csv] and modality definitions are provided in Data S1: [ModalitiesPerTraitDefinitions_EPR.csv].

### Clustering species into functional guilds by similarity in trait modalities

Species were clustered into functional guilds based on the similarity of their modalities for all eight traits. The pairwise dissimilarity of species was calculated using the function gowdis in the R package FD (Laliberté et al. 2015; R version 4.0.3). We chose Gower dissimilarity because it accepts both numerical and categorical data and handles missing values (Gower 1971). Podani’s extension was implemented to include ordinal variables (Podani 1999). Clusters were computed from the Gower dissimilarity matrix using the hclust function in the R package cluster (Maechler 2018) and plotted as a dendrogram. The cutoff for assigning functional guilds was chosen by optimizing the tradeoff between minimizing within‐group distance and maximizing between‐group distance. Code for this and subsequent analysis is available online (Dykman 2021).

### Diversity indices

Diversity was calculated for both species and functional guilds using Hill number of order 1 (Hill 1973), which is the exponential of the Shannon‐Wiener index (Jost 2006). We used this metric because it has an ecologically intuitive interpretation, accounts for varying sample size, and weighs species according to their abundance (Appendix S4). For functional diversity, we calculated Rao’s Quadratic Entropy (RaoQ), a commonly used index that considers both the relative abundance of species and the functional dissimilarity between species (Rao 1980). RaoQ was calculated using the function dbFD in the R package FD. Trends in another commonly used metric returned by dbFD, functional richness (FRic), are qualitatively similar, and are provided for comparison with other studies (Appendix S4: Fig. S1).

### Statistics

We used multinomial logistic regression to test the statistical significance of the change over time of both functional guilds and modalities within each trait. These analyses were carried out using the R package nnet (Venables and Ripley 2002). Briefly, under this model, guild or modality counts are assumed to have a multinomial distribution, with the log of the ratio of the relative abundance of each guild or modality to the relative abundance of a baseline guild or modality being a function of time. To allow for non‐monotonic trends in relative abundance, we took this function to be quadratic in time. The model was fit by maximum likelihood, and the significance of the fitted model was assessed by randomization. This involved repeatedly randomizing the samples while keeping the observation times fixed. The regression model was fit to the randomized data, and the *P*‐value was taken as the proportion of 1,000 randomizations for which the randomized deviance was less than the observed deviance. We used randomization because it is insensitive to extra‐multinomial variability due, for example, to dependence between individuals. The relationship between diversity indices and time was assessed by ordinary least squares regression with both time and its square as regressors. The significance of the fitted model was taken as the proportion of 1,000 randomizations for which the randomized *R* ^2^ value exceeded the observed *R* ^2^ value.

## Results

### Biological traits

**Fig. 2:**
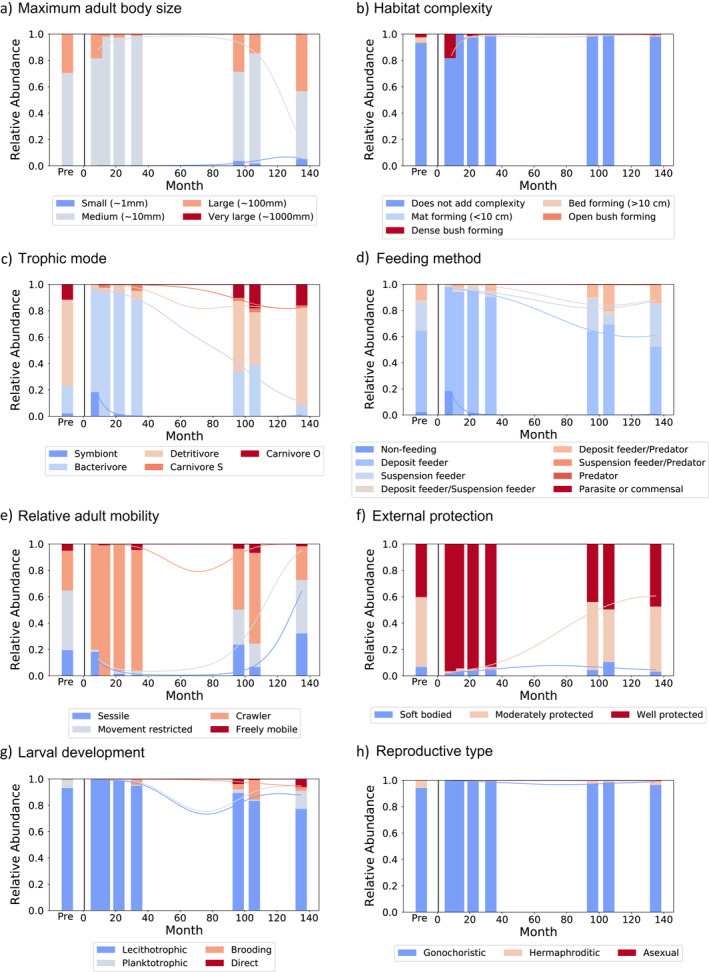
The relative abundance of modalities within the traits: (a) maximum adult body size, (b) habitat complexity, (c) trophic mode, (d) feeding method, (e) relative adult mobility, (f) external protection, (g) larval development, and (h) reproductive type. The *x*‐axis shows time in months since the 2006 eruption, with pre‐eruption samples plotted to the left of the black line. Best‐fit lines were generated by multinomial logistic regression. *P* values calculated by randomization are provided in Appendix S5: Table S1.

For most traits, the composition of modalities changed over time, often trending toward, or overshooting, the pre‐eruption state (Fig. 2). The species or taxonomic groups driving these trends typically were siboglinid tubeworms and limpets early in the time series, and a variety of worm and crustacean species toward the end (species counts per sandwich or block in Data S1: CountsPerSpeciesPerSandwich_EPR.csv; modality assignments per trait in Data S1: ModalitiesPerTraitPerSpecies_EPR.csv).

For the trait “maximum adult body size,” large organisms were most abundant both at 9 months and at 96 months or later post‐eruption (Fig. 2a). The large organisms at 9 months were siboglinid tubeworms, whereas the large organisms at 96 months and later were other polychaetes including serpulid tubeworms and *Nicomache* sp.

In the trait “habitat complexity,” bush‐forming foundation species were abundant 9 months after the eruption (Fig. 2b) and were practically absent thereafter. Bed‐forming animals peaked 22 months post‐eruption, with the colonization of *Bathymodiolus thermophilus* mussels. Most organisms throughout the time series did not form structure.

In both of the feeding ecology traits, “trophic mode” and “feeding method,” modalities changed markedly over time and trended toward the pre‐eruption states. In the trait “trophic mode,” symbiont hosts were abundant at 9 months, and bacterivores were abundant from 9 months to 33 months post‐eruption. Organisms in higher trophic levels, such as detritivores and carnivores, increased over time and were dominant at 96 months and later (Fig. 2c). For the trait “feeding method,” deposit feeders decreased in abundance around 33 months post‐eruption, while suspension feeders such as serpulid species and the barnacle *Neolepas zevinae* became most abundant at 96 months and later (Fig. 2d). Carnivores such as the polychaetes *Archinome rosacea*, *Thermiphione risensis*, *Nereis* sp., and the snail *Phymorhynchus major* were similarly most prevalent 96 months post‐eruption and later.

In the trait “relative adult mobility,” most organisms early in the time series were crawlers (Fig. 2e). Organisms with restricted movement, such as the polychaetes *Amphisamytha galapagensis*, *Nicomache* sp., and *Branchipolynoe* sp., as well as freely mobile organisms, such as amphipods, became more prominent at 96 months and later. Sessile organisms were present both early and late in the time series.

Regarding “external protection,” well‐protected organisms such as gastropods (i.e., *Lepetodrilus* spp.) and tubeworms were abundant in early samples, but decreased after 33 months (Fig. 2f). Moderately protected worms such as *Amphisamytha galapagensis* and *Archinome rosacea* increased in abundance over time, and soft‐bodied organisms were most abundant in the middle of the time series.

Within the reproductive trait “larval development,” lecithotrophic organisms were most abundant early in the time series and decreased over time, while planktotrophic and brooding development increased (Fig. 2g). There was little change in modalities within “reproductive type” (Fig. 2h).

Of the eight traits tested for significant changes over time, only trophic mode (*P* = 0.003), feeding method (*P* = 0.031), and external protection (*P* = 0.010) showed significant trends under the quadratic model (Appendix S5: Table S1).

### Functional guilds

**Fig. 3:**
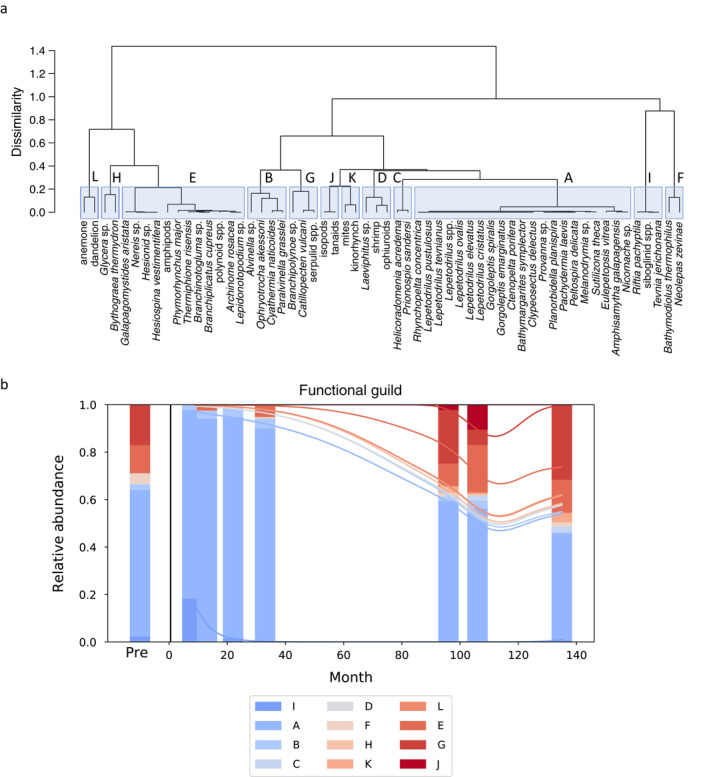
(a) A dendrogram of species dissimilarity based on modality assignments for the eight functional traits. The dendrogram was generated by hierarchical clustering using Gower dissimilarity. The *y*‐axis represents dissimilarity between species. (b) The relative abundance of functional guilds in each sample over time, with pre‐eruption data shown to the left of the black line. Note that the 96‐month sample is similar in composition to the pre‐eruption sample, which was collected 85 months after a prior eruption. Best‐fit lines were calculated by multinomial logistic regression.

The 58 species retained in our analysis were optimally clustered into 12 guilds (Table 2; Fig. 3a). Guild I, which included large, structure‐forming, non‐feeding, symbiont‐hosting tubeworms, was extremely abundant immediately after the eruption and declined within 33 months. Three guilds, A, B, and F, peaked at 22 months. Guild A included 21 deposit‐feeding, crawling bacterivore species; Guild B included deposit feeders and suspension feeders, some of which formed structure; and Guild F included suspension feeders with restricted movement, hermaphroditic reproduction, and planktotrophic larvae. Guilds E, G, and J increased over time. Guild E included moderately protected, mobile scavengers and carnivores; Guild G included sessile or restricted‐movement suspension feeders and commensals; and Guild J included well‐protected, brooding crustaceans. Time since the eruption was a significant predictor of guild composition (*P* = 0.029) (Appendix S5: Table S1), which transitioned over time toward the pre‐eruption state at 96 months and continued to change thereafter (Fig. 3b).

**Table 2:** The 12 functional guilds generated by hierarchical clustering.

Guild	*n*	Representative Species	Modalities
A	21†	*Lepetodrilus tevnianus*	medium (~10 mm); does not add complexity; bacterivore; deposit feeder; crawler; well protected; lecithotrophic; gonochoristic
B	4	*Ophryotrocha akessoni*	medium (~10 mm); does not add complexity; detritivore; deposit feeder/suspension feeder; crawler; soft bodied; lecithotrophic
C	2	*Helicoradomenia acredema*	small (~1 mm); does not add complexity; bacterivore; deposit feeder; crawler; moderately protected; lecithotrophic; hermaphroditic
D	3†	Ophiuroids	large (~100 mm); does not add complexity; carnivore S; deposit feeder; crawler; well protected; planktotrophic
E	12†	*Archinome rosacea*	medium (~10 mm); does not add complexity; carnivore O; deposit feeder/predator; crawler; moderately protected; gonochoristic
F	2	*Bathymodiolus thermophilus*	large (~100 mm); bed forming (>10 cm); detritivore; suspension feeder; movement restricted; well protected; planktotrophic; hermaphroditic
G	3†	Serpulid spp.	large (~100 mm); does not add complexity; detritivore; suspension feeder; sessile; well protected
H	2†	*Bythograea thermydron*	large (~100 mm); does not add complexity; carnivore S; predator; freely mobile; well protected; planktotrophic; gonochoristic
I	3†	*Tevnia jerichonana*	large (~100 mm); dense bush forming; symbiont; non‐feeding; sessile; well protected; lecithotrophic; gonochoristic
J	2†	Isopods	medium (~10 mm); does not add complexity; detritivore; deposit feeder; crawler; well protected; brooding; gonochoristic
K	2†	Mites	small (~1 mm); does not add complexity; carnivore O; deposit feeder; crawler; moderately protected; direct; gonochoristic
L	2†	Anemone	large (~100 mm); does not add complexity; carnivore O; suspension feeder/predator; sessile; soft bodied; planktotrophic; asexual

Notes

For each guild, a representative species is shown with its modality assignments, along with the number of species in the guild (*n*).

†The guild contains at least one group identified at a higher taxonomic level than species, and potentially contains multiple species (e.g., *Lepetodrilus* spp., siboglinid spp.).

### Diversity indices

**Fig. 4:**
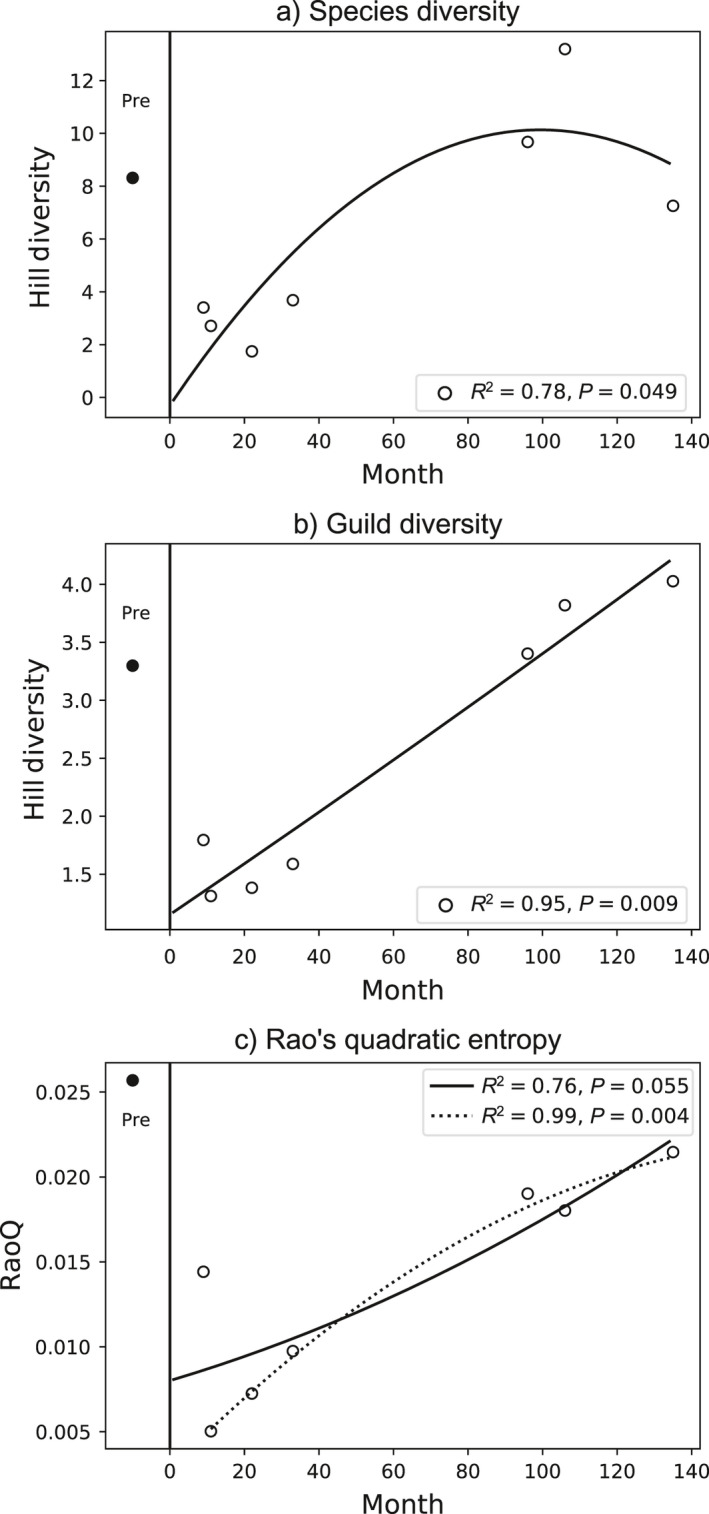
Changes in diversity over time: (a) Hill diversity (order 1) for species, (b) Hill diversity (order 1) for guilds, and (c) Rao’s quadratic entropy. For all panels, pre‐eruption data are plotted to the left of the black line. Best‐fit lines were calculated by fitting a quadratic model. In panel c, fit lines are shown both including (solid line) and excluding (dotted line) the outlier at 9 months. *P* values calculated by randomization are shown in the legend.

Both species and guild diversity increased significantly over time (*R^2^ = 0.78*, *P = 0.049* for species; *R^2^ = 0.95, P = 0.009* for guild) and eventually exceeded pre‐eruption levels (Fig. 4a, b). While species diversity reached pre‐eruption levels around 60 months and attained a maximum inflection point around 100 months, guild diversity continued to increase steadily until the final sample at 135 months. RaoQ increased more gradually and had not returned to pre‐eruption levels by the end of the time series, although this trend was not statistically significant (*R* ^2^ = 0.76, *P* = 0.055) (Fig. 4c). The sample at 9 months was an outlier with a high RaoQ compared to the other samples early in the time series, suggesting a rapid, yet temporary, return of functional diversity. When excluding the outlier at 9 months, RaoQ had a significant quadratic relationship with time (*R* ^2^ = 0.99, *P* = 0.004) and was beginning to reach an asymptote below the pre‐eruption level by the end of the time series.

## Discussion

### Testing trait‐based successional theories in deep‐sea hydrothermal vent ecosystems

Our functional analysis indicates that invertebrate communities at frequently disturbed hydrothermal vents show primary successional patterns that differ from other systems in several key ways. Importantly, the succession of function at vents differs from theory when considering traits related to size, growth, and provision of habitat (foundation species). These patterns are likely consequences of the venting flux associated with volcanic disturbance, which provides nutrients in the form of reduced sulfur that fuels the chemosynthetic‐based food web in early stages of succession.

Theory and observation in a range of ecosystems suggest early successional species are often small, due to the harsh abiotic conditions and low nutrient availability after disturbance (MacArthur and Wilson 1967, Thornton 2007), while large species establish later, grow slowly, and gradually build habitat structure that supports many other species (Tilman 1988, Hirata 1992, Teixidó et al. 2004). Our analysis indicates vent ecosystems represent an exception. Large species, primarily siboglinid tubeworms, were among the earliest colonists. Siboglinids are the largest invertebrates sampled in our time series (0.35 m for *Tevnia jerichonana*; Desbruyères et al. 2006), and they are known for their high growth rates (Lutz et al. 1994) and their role in building dense, bushy habitat structure that supports many species (Govenar and Fisher 2007). Siboglinids rely entirely on chemosynthetic endosymbiotic bacteria that use reduced sulfur to fix carbon, and accordingly their arrival corresponded with the vigorous venting following the eruption. They were gradually replaced by serpulid tubeworms (which suspension feed) over succession, which suggests the changing chemical environment of the vent field or other transitions in food supply may have driven the dramatic and rapid decrease in large‐bodied foundation species early in succession.

Despite the unusually high nutrient input in early succession at vents, the observed changes in feeding traits were consistent with established theory. As in other successional systems (Margalef 1963, Odum 1969, Boit and Gaedke 2014), patterns in feeding traits indicate a transition from high primary productivity and low food web complexity, toward greater food web complexity, more trophic levels, and a greater range of feeding strategies. Although we expected trophic diversity to recover rapidly at vents due to the high nutrient input in early succession, scavengers, carnivores, and suspension feeders only became prominent after 96 months. This indicates that the food web at vents takes longer to recover than expected, which has implications for the persistence of high‐trophic‐level species in frequently disturbed ecosystems. Prolonged recovery in feeding traits may arise because the patchy nature of vents poses dispersal challenges to some species, and because predators require the prior arrival of prey.

The significant decrease in well‐protected organisms (e.g., animals with shells and tubes) late in the time series is consistent with the expectation that external protection allows vent organisms to endure the harsh thermal and chemical conditions in early succession, and thus take advantage of the rich nutritional environment associated with disturbance. Predation is another factor that could influence changes in external protection. We observed predators to increase as succession progressed, which might be expected to result in the elimination of prey that lack external protection. However, highly mobile predators such as crabs are reported to immigrate soon after disturbance (Shank et al. 1998) and may have been important transient scavengers in the vent field in early stages of succession. These wide‐ranging adult predators would not have been captured by our sampling methods and might have been responsible for the scarcity of soft‐bodied organisms in early succession.

As observed in other patchy, island‐like systems (MacArthur and Wilson 1967, Tsuyuzaki and del Moral 1995, Thornton 2007), we expected vent organisms with brooding and direct development (i.e., no planktonic dispersal stage) to arrive later in the time series due to their limited dispersal potential (Tyler and Young 1999). According to expectation, vent animals with low dispersal potential were indeed most abundant at the end of the time series. Successional theory also suggests early colonizers are small and rapidly reproducing (i.e., “*r*‐selected” species; MacArthur and Wilson 1967) in addition to being good dispersers. Contrary to this expectation, unicellular protozoans (ciliates and foraminiferans), which might be considered classic early successional species, typically are not found in the early years of vent succession (Mullineaux et al. 2020). It is likely that the patchy distribution of vent habitat makes dispersal a dominant factor in successional outcomes compared to systems that are less island‐like. In our case, species with classic early successional traits (i.e., small body size, asexual reproduction) may not have arrived because they lacked a long‐duration planktonic dispersal stage. However, a few individuals of species with benthic and brooding development (amphipods and kinorhynchs) were present in the earliest sample. Since the nearest undisturbed site after the 2006 eruption was 6 km away, this implies that even invertebrates with dispersal limitations have the potential to travel this far within 9 months.

### Information from functional diversity indices on processes and timescales of recovery

We tracked two functional diversity metrics over time to see whether they provide additional information on processes and timescales of recovery compared to species diversity indices (Cadotte et al. 2011). Greater functional diversity means a community has a wider range of responses to perturbation and an enhanced ability to maintain structure and stability (Carr et al. 2018). Functional diversity indices are also powerful for their ability to highlight species that disproportionately enhance ecosystem functioning. For example, the relatively high RaoQ in the 9‐month sample was largely due to the functional dissimilarity between the groups *Lepetodrilus* spp. and siboglinid spp. (Fig. 3a), which were abundant at that time. This demonstrates the colonization of species with distinctive functional roles can facilitate a rapid, albeit transient, recovery of functional diversity in early succession. Most significantly, our study shows that both guild diversity and Rao’s quadratic entropy continue to increase 11 yr after catastrophic disturbance, even as species diversity was no longer increasing. This indicates that function at vents may take longer to recover than species diversity and motivates the use of alternate metrics to species diversity when assessing resilience. Moreover, our study shows that species and guild diversity increased beyond our pre‐disturbance sample, which indicates the pre‐disturbance sample did not represent a maximum assemblage of functions. This finding, along with a similar result for species diversity in a broader set of samples over the same time frame (Mullineaux et al. 2020), reinforces the importance of properly interpreting “baselines” in frequently disturbed systems.

### Applications to vent ecology and conservation

Long‐term functional analysis leads to several important insights regarding recovery and succession following volcanic disturbance at deep‐sea hydrothermal vents. First, eruptions open space for developing communities with distinct trait modalities and functional guilds, and their composition changes markedly over succession. In this conceptual framework, eruptions support a large regional pool of functions by maintaining a patchwork of vents at different stages of succession. Second, functional diversity follows different temporal patterns than species diversity, and takes longer to recover. As in other systems, it is possible that diversity in the EPR community will decline beyond our time series after reaching a peak (Connell 1978), in which case decadal volcanic disturbance at the EPR may play a role in maintaining high species and functional diversity. Finally, there was striking similarity in trait and guild composition between the pre‐eruption sample, collected 85 months after the 1991 eruption, and the samples collected 96 months after the 2006 eruption. This is a compelling indication of predictable, repeating patterns in the recovery of function over succession at this vent field. These insights motivate the expansion of long‐term monitoring programs to test trait‐based hypotheses later in succession and at hydrothermal regions with different species and conditions.

Several features of our sampling protocol limit resolution on successional processes. Colonization surfaces were deployed in series for short intervals, which did not allow a mature community to develop on any given sandwich or block. This means our sampling regime did not capture the interspecific interactions that influence succession when space is limited. Sandwiches were also deployed for variable times, which means the community on the surfaces were at various stages of maturity at recovery. However, there was always open space on recovered sandwiches, even those deployed for years, so we do not expect deployment duration led to significant exclusion of colonists. The timing of spawning events and stochastic larval supply determine the composition of settlers to some extent in all colonization experiments (Sutherland and Karlson 1977, Underwood and Chapman 2006). Because many vent invertebrates spawn continuously or asynchronously (Tyler and Young 1999), and because most sandwiches were deployed for more than a year, we expect this reduced the influence of large random recruitment events on results. Sandwiches also undersample animals with certain modalities, such as highly mobile species or large adults. As in all functional analyses, the traits used in this study may have failed to capture all subtleties in niches. Trait information for most deep‐sea species remains incomplete or low in certainty. In our case, we expect taxonomic uncertainty for species within the major abundant groups that drove patterns (e.g., siboglinid spp. and *Lepetodrilus* spp.) did not lead to uncertainty in functional interpretations because modalities for these species were well‐described, and species within each group tended to have similar modalities for the traits we examined. We expect our analysis can be updated as trait and taxonomic data become more refined.

An understanding of recovery processes in deep‐sea ecosystems has increasing practical application due to the development of seabed mining. In order to predict how endemic vent fauna will respond to anthropogenic disturbance, the patterns and drivers of succession after natural disturbance must first be understood (Boschen et al. 2013). Our data indicate modalities related to high community productivity, such as large body size, symbiont‐hosting, and structure‐forming were particularly abundant directly after the eruption. Mining at deep‐sea vents differs from natural disturbance by imposing direct damage, seabed alteration, and sediment plumes (Van Dover 2014) without necessarily promoting new venting. Thus, recovery from anthropogenic disturbance will likely depend on the timing and location of mining disturbance in the vent field and whether mining stimulates new venting. The combination of long‐term colonization data and functional traits is an important advance for assessing resilience and developing successional theory that allows comparison between ecosystems. Frequent disturbance, atypical stress and nutrient conditions, and unique resident fauna make hydrothermal vents a compelling new system in which to expand successional research and broaden existing theory.

## Supporting information

Appendix S1

Appendix S2

Appendix S3

Appendix S4

Appendix S5

Data S1

## Data Availability

Dates and locations of colonization sampler deployments and recoveries (Mullineaux 2020, 2020b) are available from the Woods Hole Open Access Server (WHOAS) repository at https://doi.org/10.26008/1912/bco‐dmo.733210.3. Counts of colonists collected from colonization surfaces are provided in Data S1 (CountsPerSpeciesPerSandwich_EPR.csv) and are a subset of Mullineaux (2020, 2020a) from the Woods Hole Open Access Server (WHOAS) repository at https://doi.org/10.26008/1912/bco‐dmo.733173.2. Modality assignments for each functional trait are provided in Data S1: ModalitiesPerTraitPerSpecies_EPR.csv and in Dykman et al. (2021) on the Woods Hole Open Access Server (WHOAS) repository at http://doi.org/10.26008/1912/bco‐dmo.844993.1. Scripts used for this analysis (Dykman 2021) are provided on Zenodo: http://doi.org/10.5281/zenodo.4625160.
