# Supplementary material for: Functional traits provide new insight into recovery and succession at deep‐sea hydrothermal vents
Source: Ecology. 2021 Jul 2;102(8):e03418. doi: 10.1002/ecy.3418 (PMC8459237; doi:10.1002/ecy.3418)
Supplement: Supplementary file 1 — Appendix S1 [file ECY-102-e03418-s006.pdf]

Supporting Information. Dykman, L.N., S.E. Beaulieu, S.W. Mills, A.R. Solow, and L.S. Mullineaux. 2021. Functional traits provide new insight into recovery and succession at deep-sea hydrothermal vents. Ecology.

Appendix S1. Colonization Surface Design and Deployment

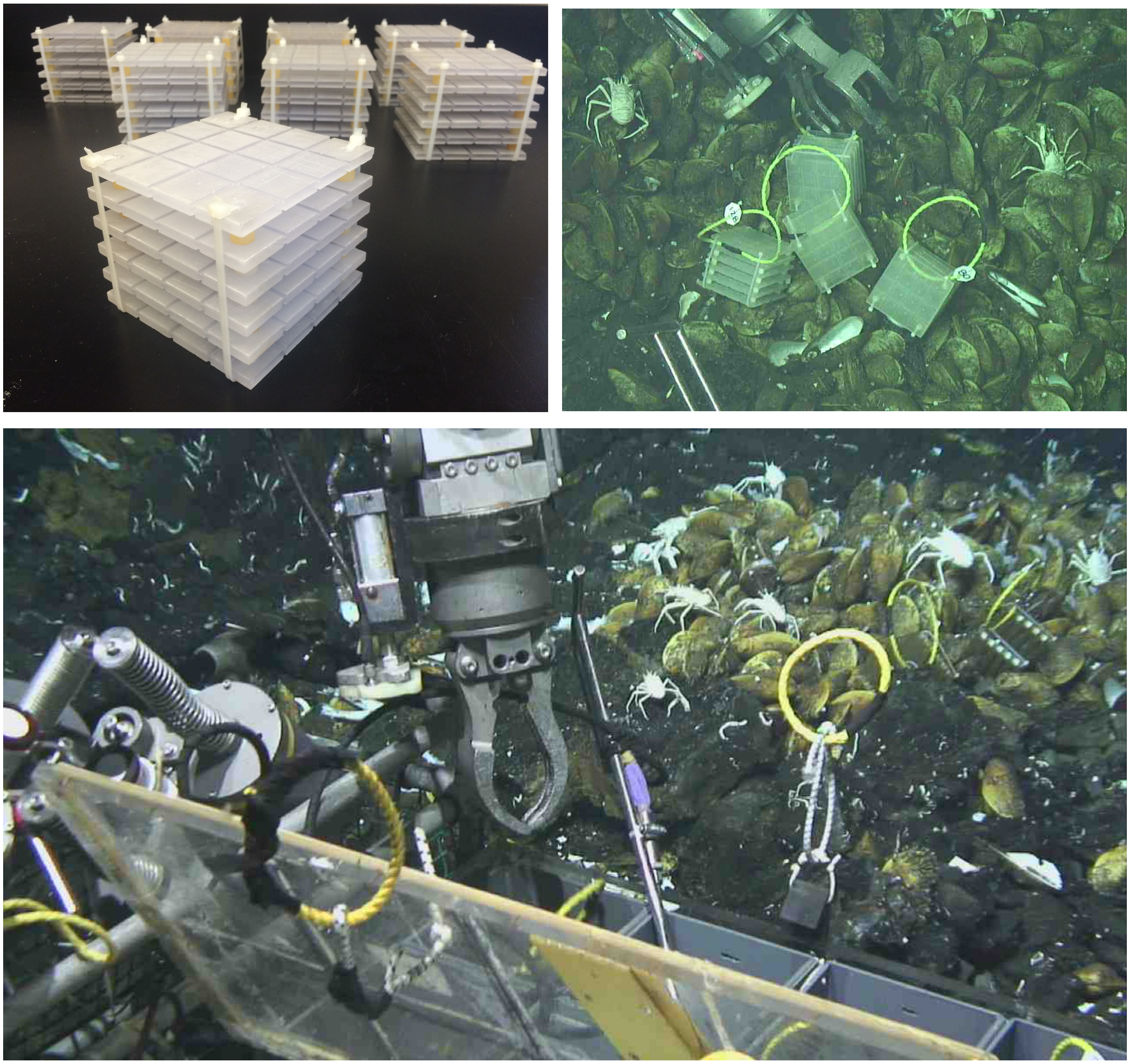

Figure S1. Deployment of "sandwich" colonization surfaces using the manipulator arm of *DSV Alvin* and a biobox. Each sandwich was constructed of six stacked 10 cm x 10 cm x 0.7 cm roughened Lexan sheets separated by 1 cm spacers. Counts of colonists on each Lexan sheet were pooled for data entry, so each sandwich constituted a single sample in analysis.
