# Supplementary material for: Functional traits provide new insight into recovery and succession at deep‐sea hydrothermal vents
Source: Ecology. 2021 Jul 2;102(8):e03418. doi: 10.1002/ecy.3418 (PMC8459237; doi:10.1002/ecy.3418)
Supplement: Supplementary file 2 — Appendix S2 [file ECY-102-e03418-s003.pdf]

Supporting Information. Dykman, L.N., S.E. Beaulieu, S.W. Mills, A.R. Solow, and L.S. Mullineaux. 2021. Functional traits provide new insight into recovery and succession at deep-sea hydrothermal vents. Ecology.

Appendix S2. Genetic analysis of a subset of individuals from the groups *Lepetodrilus* spp. and siboglinid spp.

Individuals within the groups *Lepetodrilus* spp. and siboglinid spp. were too young to identify to species. Subsets of these individuals from months 9, 22, and 33 were sent to the Canadian Center for DNA Barcoding (CCDB) in July of 2018 for genetic sequencing. Sequencing targeted the mitochondrial cytochrome oxidase I (COI) gene, using the CCDB Next Generation Sequencing (NGS-FT) protocol. Specimen and sequence data were uploaded to the Barcode of Life DataSystems (BOLD) and queried against the BOLD COI full database. Of the 45 individuals of *Lepetodrilus* spp. sent for sequencing, all yielded sequences. Thirteen were matched to Barcode Index Numbers (BINs), which are operational taxonomic units, defined by widely used clustering algorithms. These closely correspond to species and reflect high levels of identification confidence. Of the specimens assigned to BINs, 11 were *Lepetodrilus tevnianus* and 2 were *Lepetodrilus elevatus*. Of the sequences not assigned a BIN, 19 were matched to *L. tevnianus*, 12 were matched to *L. elevatus*, and 1 was matched to *L. aff. galriftensis* (a conservative identification that has been grouped in the same BIN as *L. elevatus*) based on the BOLD COI full database. Individuals identified as *L. elevatus* were only present in the samples from 22 and 33 months, which agrees with observations that *L. elevatus* typically arrive later in succession than *L. tevnianus*. Of the 40 individuals of siboglinid spp. sent for sequencing, 38 yielded sequences. None were assigned BINs, but 35 were matched to *Tevnia jerichonana* and 3 were matched to *Oasisia alvinae* using the BOLD COI full database. *T. jerichonana* is the tubeworm species normally observed earliest in succession at the EPR. These data are publicly available using the project search "EPRBE" in:

http://www.boldsystems.org/index.php/Public_BINSearch?searchtype=records
