# Supplementary material for: Functional traits provide new insight into recovery and succession at deep‐sea hydrothermal vents
Source: Ecology. 2021 Jul 2;102(8):e03418. doi: 10.1002/ecy.3418 (PMC8459237; doi:10.1002/ecy.3418)
Supplement: Supplementary file 3 — Appendix S3 [file ECY-102-e03418-s001.pdf]

Supporting Information. Dykman, L.N., S.E. Beaulieu, S.W. Mills, A.R. Solow, and L.S. Mullineaux. 2021. Functional traits provide new insight into recovery and succession at deep-sea hydrothermal vents. Ecology.

Appendix S3. Rationale for Trait Selection and Suggested Updates to sFDvent Database

Of the thirteen recommended traits in sFDvent (Chapman et al. 2019), we did not use: 1) gregariousness, 2) foundation species, 3) abundance, 4) chemosynthesis-obligate, 5) depth, 6) zonation from a vent, 7) nutritional source, and 8) substratum. Traits 1, 3, and 6 were omitted because we considered them "emergent" rather than "inherent" traits. In other words, these were seen as population-level patterns that arise due to the traits of species, not traits themselves (Garnier et al. 2015). Trait 2) was omitted because it was redundant with "habitat complexity," as foundation species by definition form structure. Traits 5) and 8) were omitted because our study took place at a single site with a depth of ~2,500 meters and basalt substrate. Our trait "feeding method" (taken from the Biological Traits Information Catalogue (BIOTIC), MarLIN 2006) is similar to 7), however our modalities provide more detail as to the animal's feeding mechanism and are consistent with terminology used in other invertebrate trait studies. Terminology for this trait varies between studies; it is known as "functional feeding group" or "feeding guild" for freshwater invertebrates (Ding et al. 2017), "feeding position" in Veríssimo et al. (2017), "feeding mode" in Bolam et al. (2016), and "feeding type" in the online polychaete trait database Polytraits (Faulwetter et al. 2014). We used the "trophic mode" trait in sFDvent, however we added the modality "symbiont" to account for animals that have no mouths or guts and do not feed. We suggest that such species are technically not bacterivores, as they are categorized in sFDvent. For "relative adult mobility," we used the same modalities as in sFDvent, but assigned names to the numerical modalities based on other trait databases such as BIOTIC (MarLIN 2006). For example, mobility level "1" in sFDvent became "sessile," and mobility level "2" became "crawler."

Table S1. Cases in which our trait modality assignments deviated from the sFDvent database. Our recommended updates are shown with citations beside the original assignments from sFDvent.

Trait	ID	Taxon Aphia ID	sFDvent Assignment	Suggested Update	Citation
**MAXIMUM ADULT BODY SIZE**	*Helicoradomenia acredema*	395145	Medium (~10mm)	Small (~1mm)	Length up to 3.5mm.[1]
**MAXIMUM ADULT BODY SIZE**	*Eulepetopsis vitrea*	449958	Large (~100mm)	Medium (~10mm)	Shell length up to 17mm.[1]
**MAXIMUM ADULT BODY SIZE**	*Laeviphitus* sp.	137922	Medium (~10mm)	Small (~1mm)	Shell length up to 1.8mm for *Laeviphitus desbruyeresi*.[1]
**MAXIMUM ADULT BODY SIZE**	*Melanodrymia* sp.	449912	Medium (~10mm)	Small (~1mm)	Shell diameter up to 3.5mm.[1]
**MAXIMUM ADULT BODY SIZE**	*Rhynchopelta concentrica*	450018	Large (~100mm)	Medium (~10mm)	Shell length up to 13 mm.[1]
**MAXIMUM ADULT BODY SIZE**	*Sutilizona theca*	450019	Medium (~10mm)	Small (~1mm)	Shell length up to 2.4 mm.[1]
**MAXIMUM ADULT BODY SIZE**	*Bathymodiolus thermophilus*	183000	Very large (~1000mm)	Large (~100mm)	Shell length up to 18.4 cm.[1]
**MAXIMUM ADULT BODY SIZE**	*Catillopecten vulcani*	391784	Large (~100mm)	Medium (~10mm)	Up to 17 mm.[1]
**MAXIMUM ADULT BODY SIZE**	*Archinome rosacea*	333028	Large (~100mm)	Medium (~10mm)	Up to 13 mm long, 5.8 mm wide.[1]
**MAXIMUM ADULT BODY SIZE**	*Thermiphione risensis*	1288749	Large (~100mm)	Medium (~10mm)	Up to 11 mm in length, width 6 mm including chaetae.[1]
**MAXIMUM ADULT BODY SIZE**	*Lepidonotopodium* sp.	182913	Large (~100mm)	Medium (~10mm)	Maximum observed 35 mm in length, 18 mm in width for *Lepidonotopodium fimbriatum*.[1]
**RELATIVE ADULT MOBILITY**	*Helicoradomenia acredema*	395145	Movement restricted	Crawler	Expert opinion. No evidence this species is restricted to crevices, tubes, or burrows at our sites.
**RELATIVE ADULT MOBILITY**	*Catillopecten vulcani*	391784	Crawler	Movement restricted	Byssally attached in diffuse venting areas.[1]
**RELATIVE ADULT MOBILITY**	shrimp	106674	Crawler	Freely mobile	Expert opinion. Shrimp have the ability to swim in the water column.
**HABITAT COMPLEXITY**	*Catillopecten vulcani*	391784	NA	Does not add	Expert opinion.
**HABITAT COMPLEXITY**	*Alvinella sp.*	336171	Burrow forming	Mat forming (<10 cm)	Expert opinion. This species forms papery tubes. Our site consist of hard basalt and sulfide deposits, so animals do not burrow.
**HABITAT COMPLEXITY**	*Nicomache* sp.	129357	Open bush forming	Does not add	Expert opinion. *Nicomache* species never observed to form structure at our sites.
**HABITAT COMPLEXITY**	*Paralvinella grasslei*	330308	Burrow forming	Does not add	Expert opinion. Form tubes, but usually in crevices, so do not contribute much structure.
**HABITAT COMPLEXITY**	*Prionospio sandersi*	558842	NA	Does not add	Expert opinion.
**HABITAT COMPLEXITY**	*Neolepas zevinae*	535261	NA	Open bush forming	Expert opinion.
**TROPHIC MODE**	*Riftia pachyptila*	266010	Bacterivore	Symbiont	We added Symbiont as a modality in TROPHIC MODE because animals without mouths or guts technically do not feed on bacteria.
**TROPHIC MODE**	siboglinid spp.	129096	Bacterivore	Symbiont	We added Symbiont as a modality in TROPHIC MODE because animals without mouths or guts technically do not feed on bacteria.
**TROPHIC MODE**	*Tevnia jerichonana*	266028	Bacterivore	Symbiont	We added Symbiont as a modality in TROPHIC MODE because animals without mouths or guts technically do not feed on bacteria.

[1]Desbruyères, Segonzac & Bright (Eds.) 2006.

References

Bolam, S.G. et al. (2016) Application of biological traits to further our understanding of the impacts of dredged material disposal on benthic assemblages. *Mar Pollut Bull* 105:180-192

Chapman, A.S.A. et al. (2019) sFDvent: A global trait database for deep-sea hydrothermal-vent fauna. *Global Ecol Biogeogr* 00:1–14

Desbruyères, D., Segonzac, M., & Bright, M. (Eds.). (2006) *Handbook of deep-sea hydrothermal vent fauna* (Vol. 18, pp. 1-544). Land Oberösterreich, Biologiezentrum der Oberösterreichische Landesmuseen

Ding, N. et al. (2017) Different responses of functional traits and diversity of stream macroinvertebrates to environmental and spatial factors in the Xishuangbanna watershed of the upper Mekong River Basin, China. *Sci Total Environ* 574:288–299

Faulwetter, S. et al. (2014) *Polytraits*: A database on biological traits of marine polychaetes. *Biodiversity Data Journal* 2:e1024

Garnier, E., Navas, M.L., and Grigulis, K. Plant Functional Diversity: Organism traits, community structure, and ecosystem properties. Ch 2: Trait-based ecology: definitions, methods, and a conceptual framework. Oxford, 2015.

MarLIN, 2006. *BIOTIC - Biological Traits Information Catalogue.* Marine Life Information Network. Plymouth: Marine Biological Association of the United Kingdom. [Cited Nov 5 2019] Available from www.marlin.ac.uk/biotic

Veríssimo, H. et al. (2017) Comparison of thermodynamic-oriented indicators and trait-based indices ability to track environmental changes: Response of benthic macroinvertebrates to management in a temperate estuary. *Ecol Indic* 73:809–824
