# Supplementary material for: Functional traits provide new insight into recovery and succession at deep‐sea hydrothermal vents
Source: Ecology. 2021 Jul 2;102(8):e03418. doi: 10.1002/ecy.3418 (PMC8459237; doi:10.1002/ecy.3418)
Supplement: Supplementary file 4 — Appendix S4 [file ECY-102-e03418-s005.pdf]

Supporting Information. Dykman, L.N., S.E. Beaulieu, S.W. Mills, A.R. Solow, and L.S. Mullineaux. 2021. Functional traits provide new insight into recovery and succession at deep-sea hydrothermal vents. Ecology.

Appendix S4. Diversity indices

Diversity indices were calculated for both species and functional guilds using Hill's diversity number of order 1 (Hill 1973). Hill diversity (N_a) is a universal diversity equation that simplifies to relatives of three common diversity indices depending on the order a (Eq. 1):

1) $N_a = (\sum_{i=1}^{S} p_i^a)^{1/(1-a)}$

For this and all following equations, let S be the number of species in a sample and p_i be the proportion of species i in the sample. The three orders of Hill diversity differ in how they weigh species abundances: N_0 measures richness, so gives more weight to rare species; N_1 is related to the Shannon-Wiener index of diversity (Shannon 1948) and weights species according to their abundance; N_2 is related to the Simpson dominance index (Simpson 1949) and weights abundant species more heavily. We chose to focus on Hill of order 1 (Eq. 2) because it takes into account sample size and weights species according to their abundance. Note, N_a is undefined when $a = 1$, but by taking the limit as a approaches 1, the equation becomes the exponential of Shannon-Wiener diversity index (Eq. 3):

2) $N_1 = exp(-\sum_{i=1}^{S} p_i ln p_i)$

3) $H' = -\sum_{i=1}^{S} p_i ln p_i$

By taking the exponential of Shannon, Hill diversity converts the index into its "numbers equivalent." In other words, it scales the index to reflect the expected numbers of species in the sample. In addition to being more ecologically intuitive (unlike the raw diversity indices which lie on an arbitrary scale), numbers equivalents also maintain intuitive mathematical properties (Jost, 2006). For example, using Hill numbers, a community with equal numbers of five species will have half the diversity of a community containing equal numbers of ten species.

Rao's quadratic entropy (RaoQ) is an index commonly used in biological trait analysis (Eq. 4). It considers both the abundance of species, and the average functional dissimilarity d_{ij} between two species i and j, without replacement (Rao 1980).

4) $\text{RaoQ} = \sum_{i=1}^{S} \sum_{j=1}^{S} d_{ij} p_i p_j$

RaoQ accepts any dissimilarity metric where d_{ij} is a number between 0 and 1. When $d_{ij} = 1$ for all $i \neq j$ (meaning all species are functionally distinct) RaoQ simplifies to the Simpson diversity index (Eq. 5), which is the compliment of the Simpson dominance index (Botta-Dukát 2005).

5) $SD = 1 - \sum_{i=1}^{S} p_i^2$

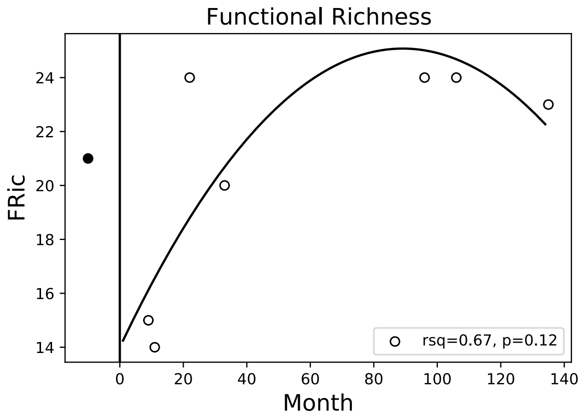

Figure S1. Trends in functional richness (FRic) over time, calculated by the function *dbFD* in the R package FD. FRic is often calculated as the convex hull volume of traits in the community (Villéger et al. 2008). In our case, FRic was calculated as the number of unique trait value combinations in the community because our trait data only included categorical and ordinal traits (Laliberté et al. 2015). This index reached pre-eruption levels faster than RaoQ, but otherwise showed similar patterns, and trends were not statistically significant (rsq = 0.67, p = 0.12). For analysis, we examined RaoQ rather than FRic because RaoQ is mathematically related to standard species diversity indices, is less sensitive to the number of species in a sample, and accounts for the relative abundance of species.

References

Botta-Dukát, Z. (2005) Rao's quadratic entropy as a measure of functional diversity based on multiple traits. *J Veg Sci* 16:533–540

Hill, M.O. (1973) Diversity and Evenness: A Unifying Notation and Its Consequences. *Ecology* 54:427–432

Jost, L. (2006) Entropy and diversity. *Oikos* 113:363–375

Laliberté, E. et al. (2015) FD: measuring functional diversity from multiple traits, and other tools for functional ecology. R Package: Version 1.0-12

Rao, C.R. (1980) Diversity and dissimilarity coefficients: A unified approach. *Theor Popul Biol* 21:24–43

Shannon, C.E. (1948) A Mathematical Theory of Communication. *Bell System Technical Journal*. 27(3):379–423

Simpson, E.H. (1949) Measurement of Diversity. *Nature* 163(4148):688–688.

Villéger, S. et al. (2008) New multidimensional functional diversity indices for a multifaceted framework in functional ecology. *Ecology* 89(8):2290-2301.
