# Supplementary material for: Functional traits provide new insight into recovery and succession at deep‐sea hydrothermal vents
Source: Ecology. 2021 Jul 2;102(8):e03418. doi: 10.1002/ecy.3418 (PMC8459237; doi:10.1002/ecy.3418)
Supplement: Supplementary file 5 — Appendix S5 [file ECY-102-e03418-s004.pdf]

Supporting Information. Dykman, L.N., S.E. Beaulieu, A.R. Solow, S.W. Mills, and L.S. Mullineaux. 2021. Functional traits provide new insight into recovery and succession at deep-sea hydrothermal vents. Ecology.

Appendix S5. Statistics for Guild and Modality Composition

Table S1. Results of multinomial logistic regression for functional guilds and traits. Deviance values and degrees of freedom "df" were calculated by the function *multnom* in the R (Version 3.4.3) package "nnet" (Venables and Ripley 2002). Chi-squared values were calculated using the function *lrtest* in the package "lmtest" and comparing the observed multinomial model to a null model with no regressors. P values were calculated by producing 1,000 randomizations of the trait abundance data while holding the time identifier constant. Statistically significant values, indicated in bold with an asterisk (*), were determined at a cutoff of $p < 0.05$.

TRAIT	BASELINE	Deviance	df	Chisq	p
**MAXIMUM ADULT BODY SIZE**	Small (~1mm)	1777	6	2322.5	0.427
**HABITAT COMPLEXITY**	Does not add complexity	646	9	3290.8	0.492
**TROPHIC MODE**	Symbiont	1132	12	9398.6	**0.003***
**FEEDING METHOD**	Non-feeding	1327	18	6536.9	**0.031***
**RELATIVE ADULT MOBILITY**	Sessile	2644	9	4914.2	0.166
**EXTERNAL PROTECTION**	Soft bodied	315	6	5219.0	**0.010***
**LARVAL DEVELOPMENT**	Lecithotrophic	554	9	1253.2	0.124
**REPRODUCTIVE TYPE**	Gonochoristic	129	6	102.3	0.512
**FUNCTIONAL GUILD**	A	1390	33	8489.6	**0.029***

References

Venables, W.N. and Ripley, B.D. (2002) Modern Applied Statistics with S. Fourth Edition. Springer, New York, USA.
