# Supplementary material for: Functional traits provide new insight into recovery and succession at deep‐sea hydrothermal vents
Source: Ecology. 2021 Jul 2;102(8):e03418. doi: 10.1002/ecy.3418 (PMC8459237; doi:10.1002/ecy.3418)
Supplement: Supplementary file 6 — Data S1 [file ECY-102-e03418-s002.zip › MetadataS1.pdf]

Supporting Information. Dykman, L.N., S.E. Beaulieu, S.W. Mills, A.R. Solow, and L.S. Mullineaux. 2021. Functional traits provide new insight into recovery and succession at deep-sea hydrothermal vents. Ecology.

Data S1

Functional trait modality assignments and counts per species for hydrothermal vent invertebrates on colonization surfaces in a long-term study following volcanic disturbance at 9°50'N on the East Pacific Rise

Authors

Lauren N. Dykman
Woods Hole Oceanographic Institution
Redfield 120 (MS# 34) 266 Woods Hole Rd. Woods Hole, MA 02543
ldykman@whoi.edu

Stace E. Beaulieu
Woods Hole Oceanographic Institution
Redfield 120 (MS# 34) 266 Woods Hole Rd. Woods Hole, MA 02543
sbeaulieu@whoi.edu

Susan W. Mills
Woods Hole Oceanographic Institution
Redfield 120 (MS# 34) 266 Woods Hole Rd. Woods Hole, MA 02543
smills@whoi.edu

Andrew R. Solow
Woods Hole Oceanographic Institution
Clark (MS# 41) 266 Woods Hole Rd. Woods Hole, MA 02543
asolow@whoi.edu

Lauren S. Mullineaux
Woods Hole Oceanographic Institution
Redfield 120 (MS# 34) 266 Woods Hole Rd. Woods Hole, MA 02543
lmullineaux@whoi.edu

File list (files found within DataS1.zip)

CountsPerSpeciesPerSandwich_EPR.csv
ModalitiesPerTraitDefinitions_EPR.csv
ModalitiesPerTraitPerSpecies_EPR.csv

Description

CountsPerSpeciesPerSandwich_EPR.csv – This data sheet provides counts of colonist species and morphogroups on the 30 post-eruption sandwiches (at P-vent) and the 6 pre-eruption blocks (at East Wall) near 9°50'N on the East Pacific Rise (EPR) used in this analysis. Recovery date (months since January 2006 eruption), sample identifier, and recovery temperature (degrees C) are presented for each sample. These are a subset of the full colonization time series available at https://doi.org/10.26008/1912/bco-dmo.733173.2

ModalitiesPerTraitDefinitions_EPR.csv – This data sheet provides a definition, citation, and type (ordered categorical or categorical) for each modality within each trait. When applicable, definitions are directly drawn from the cited database or publication. In some cases, examples and/or modifications to the trait definition from the original citation are included with the definition.

ModalitiesPerTraitPerSpecies_EPR.csv – This data sheet provides modality assignments for each species or morphogroup for each of the twelve traits included in this analysis, as well as the functional guild assignment of each species determined by hierarchical clustering. Citations for each modality assignment are entered in a column to the right of the modality assignment. NA indicates no modality information was available for that trait. "Expert Opinion" indicates the modality assignment came from the direct observation of one of the co-authors. Species and morphogroups were matched to equal or lowest level taxon in the World Register of Marine Species (WoRMS) (scientificNameID). These data are available at the Biological and Chemical Oceanography and Data Management Office (BCO-DMO) http://doi.org/10.26008/1912/bco-dmo.844993.1.
